# Attitudes of Hungarian asthmatic and COPD patients affecting disease control: empirical research based on Health Belief Model

**DOI:** 10.3389/fphar.2013.00135

**Published:** 2013-11-13

**Authors:** Judit Simon

**Affiliations:** Health Marketing Research Centre, Institute of Marketing and Media, Corvinus University of BudapestBudapest, Hungary

**Keywords:** medication adherence, chronic diseases, health belief model, asthma, COPD

## Abstract

**Introduction:** Patient non-adherence to treatment is a major problem across most chronic diseases. In COPD and asthma treatments it is a complex issue because people need to make behavioral and lifestyle changes while taking medications. Poor adherence results in increased rates of morbidity and mortality, more frequent hospitalizations, and ultimately higher healthcare expenditures.

**Materials and methods:** The objective of the study was to assess asthmatic and COPD patient's attitudes toward adherence in Hungary. Health Belief Model was used to help explain reasons of non-adherence. The results of the study should provide additional support to understanding health-related behaviors and to developing health related programs enhancing adherence of asthmatic and COPD patients.

145 diagnosed COPD patients and 161 diagnosed asthmatic patients were involved in 6 pulmonary centers. The questions were designed to measure Health Belief Model dimensions A 1–5 point verbal Likert scale was used. As a second stage, the answers were compared with the registered patient's personal health data available in pulmonary center's documentation. The data was analyzed using SPSS software.

**Results:** More than 32% of patients are very interested in new asthma or COPD research results, but their main information source is physician. The trust toward the physician is very high. Patients accept treatments and rarely ask questions. Respondents are cooperative but sometimes fail to follow therapeutic recommendations. There is no willingness to join self-help groups or associations.

**Discussion:** The paternalistic approach was generally accepted, moreover expected by the patients from the physicians. It is important to train patients, increase their self-efficacy, responsibility and involve them into self-management programs. Both physicians and patients should be trained how to communicate—this approach can lead to increased understanding and better adherence.

## Introduction

WHO estimates that minimum 300 million people are affected by asthma globally, while annual mortality is estimated to be 250,000 (Global Initiative, 2012). Disease prevalence is on the increase in most countries, mainly among children. It is estimated that by 2025 the number of patients might reach 400 million, despite the availability of effective and modern treatment methods in more and more countries.

In Hungary, the prevalence of asthma is estimated to be around 2–15%. The number of patients suffering from asthma in the lungs increases year after year. According to the Hungarian Statistical Bureau (KSH) the number of known and registered patients was 128,809 in 2000, and as high as 223,376 in 2007 and 272883 in 2012 (Böszörményi et al., 2013).

Asthma is considered to be an under-diagnosed disease (Nathan et al., 2004). As symptoms are non-specific, sometimes the physician and the patient misinterpret the symptoms, considering them as originating from infection, and it is treated as such, and that is why late diagnosis is frequent.

The pathophysiology, diagnostics, clinical treatment, therapeutic management and prevention of asthma is summed up in every detail in an international consensus called GINA (2012, Global Initiative for Asthma, www.ginasthma.org).

COPD is characterized by a slowly and gradually progressing constriction of the airway and breathing dysfunction, which are mostly irreversible (that is unlike in the case of asthma this constriction in the airway will not be resolved but maximum mitigated by bronchodilator or other treatment). In developed industrial countries 4–7% of the population are affected by this disease, and the prevalence is rising globally. According to a WHO estimate made in 2007, 210 million people suffer from COPD.

Like asthma, COPD is also under-diagnosed, since the patients are unaware of their risk factors and adapt slowly to their changes. If the current tendency continues, it is going to be the third death cause by 2030 (with a mortality rate of 8.6%); although COPD will move up “only” one level in the ranking list, but in the percentage ratios the increase in COPD mortality will be probably the highest (3.6%).

Like for asthma, a global initiative also exists in the case of COPD [GOLD (2013)—“Global Initiative for Chronic Obstructive Lung Disease”] which was formed in 1998 and has constantly published updated guidelines related to the diagnostics and treatment of this disease (www.copdgold.com).

International and Hungarian studies (Bergquist and Crompton, 2001; Herjavecz et al., 2003) led to the conclusion that despite the state-of-the-art medicinal treatment options currently available, the treatment goals have not been met for most of the patients. Only 20–30% of the patients are controlled and approx. the same ratio (20–30%) are partially controlled, that is the number of uncontrolled patients based on the clinical symptoms can be estimated as representing 40–60%.

This high level of non-control can be explained partly by the fact that patient adherence to medications is inadequate However, adherence is not a stand-alone phenomena and should be investigated in the context of doctor-patient communication (Zolnierek and DiMatteo, 2009).

Studies indicate that many patients do not take the medication as prescribed (Rand et al., 1992; Bosley et al., 1995; Clark et al., 1999) and non-adherence to treatment is a cause of mortality and morbidity in asthma.

Because of the magnitude of non-adherent behavior in asthma and COPD there is an interest to find out:

- whether patient adherence in the case of asthma and COPD will be influenced by the patients' perception and opinion on the severity of illness
- which are the main factors influencing the patient adherence and the attitude toward therapy
- which are the main barriers for better patient adherence
- which are the opportunities of doctor-patient communication enhancing patient adherence.

## Materials and methods

### The theoretical framework of the research: the health belief model

Fundamentally, the purpose of this model is to explain the choice of health behavior and the probability of choosing a healthcare related form of behavior (Rosenstock, 1974; Becker et al., 1978; Kotler and Clarke, 1987).

The model is built on the general assumption that people are willing to do something for the assessment, screening and control of their health condition and disease prevention if they consider themselves susceptible to a disease and consider the disease severe and threatening, and if they think that there are certain actions with which the risk of catching that disease can be reduced and that the time and/or financials spent on such action is compensated for by the outcome resulting from not catching the disease.

The basic variable categories of the model:

#### Perceived susceptibility

Subjective risk connected to a given health-related circumstance or disease as perceived by the individual. In the case of physician-diagnosed diseases it contains factors related to susceptibility associated with the diagnosis, the personal conviction meaning the probability attributed by the individual to his/her recovery and in general with the disease.

#### Perceived severity

Emotions describing how severe the individual perceives the condition associated with the given disease, including health risks related to hospital and other medical treatment (pain, death, disability), and social risks as well (working ability, family life, social relationships).

#### Perceived benefits

A benefit can be attributed to the action based on our perception of the action that can be done in relation to the susceptibility to the given disease: if we believe that effective preventive measures can be taken, then there exists a perceived health benefit for us. A different type of benefit may also be associated with a preventive action, for instance quitting smoking can mean financial savings too.

#### Perceived barriers

A disease preventing action might have perceived disadvantages, too, which may prevent the completion of the recommended action. There might be several preventive factors that often are not consciously described by the patient, but which may be felt regarding an action, for instance that it is certainly expensive, or that the action could be dangerous, there may be adverse effects, and it could be unpleasant, painful, time-consuming and other similar anxieties. According to Rosenstock (1974) it is the effect of benefits following the joint consideration of susceptibility and perceived ‘severity’ that gives the energy and urge for the desired action.

#### Cues to actions

Several different opinions have evolved regarding these factors that trigger action. Such a factor could be some kind of a physical condition change or an impact from the environment, or perhaps an effect communicated by the media. Few empirical studies have been conducted regarding these cues and their impact.

#### Other variables

Socio-demographic and structural variables may influence individual perception and this might have an indirect impact on health related behavior.

#### Self efficacy

The conviction that the action, with which good results can be achieved, can be performed successfully (Bandura, 1977; Rosenstock et al., 1988) suggested that the original model should be supplemented to include the belief in self efficiency, action efficacy, as a separate construction.

Patient adherence to asthma therapy can be influenced by several factors, which are complex and involve psychological, social and medical issues (Clark et al., 1999). Based on the fact that few data are available to show the effect of psychological factors on patient adherence to asthma treatment (Clark et al., 1999) and patient adherence is influenced by the attitude of the patients, it is suggested to apply psychological models to explain the connection between adherence and attitude.

One of the best known and recommended psychological models is the Health Belief Model, it was originally formulated to explain specific forms of behavior related to health, in which predominantly cognitive variables had been included. In order to make someone change (mostly through a single action) his/her health-related behavior in this perspective, usually threats were applied, attempting to influence the intellect by explaining what threats the individual would face if he/she did not participate in the given action. However, given the changed problems, the method of communication should also be reasonably changed, and encouragement is just as important as the explanation of the risks. This means explaining the benefits that the individual will enjoy once he/she managed to change his/her behavior, and this communication could involve many emotional elements, too.

The HBM has provided a useful theoretical framework for investigators of the cognitive determinants of a wide range of behaviors for more than thirty years. The model's common sense constructs are easy for non-psychologists to assimilate and can be readily and inexpensively operationalized in self-report questionnaires (Abraham and Sheeran, 2008).

### Empirical study

Similarly to international studies using the Health Belief Model (Clark, 1999; Howell, 2008), we used this model as a framework in our empirical research, too, as the questionnaire was formulated based on the model variable.

The primary objective of this empirical research has been the study of the disease related behavior and perception of asthmatic and COPD patients based on the HBM. In our study, the subjective judgment and opinion of the patients were compared with the data of paper-based documentation (source documentation) available in healthcare institutions.

Our study aims included exploring whether the patients preferred the unconditional acceptance and performance of the recommended therapy or and acceptance and adherence based on agreement. Our goal was to find the possibilities that are available for more efficient patient information and patient education, as well as those that facilitate the dialogue between the chronic patient and his/her treatment physician.

### Research methodology

The research included survey among asthmatic and COPD patients with a self-administered questionnaires. Questionnaires were asked in six out of the seven pulmonary centers of the country, and the target was 30 asthmatic and 30 COPD patient questionnaires per center. Finally, we gathered 163 completed (and evaluable) asthmatic and 147 completed (and evaluable) COPD questionnaires. We chose the pulmonary centers because we can find there the chronic patients and the best administered dossiers of patient data. The doctors in the centers were ready to help with additional information when it was needed to the comparison of registered and self-administered survey data.

The sampling method was convenience sampling, we could not use a statistical method and the quotas in each center were equal, they were not based on the prevalence data in the population. We chose this method because of the lack of regional prevalence data. This has been a retrospective, quantitative, single cross-sectional study, without follow-up on the patient and without involving a later prospective section. The target population consisted of men and women aged 18 and above with registered bronchial asthma or COPD diagnosis, receiving care in the given lung center.

The contents of the questionnaires practically matched for the two patient groups, the differences originated mainly from the different ethiologies of the diseases and the names of the diseases. For easier distinction, the questionnaires of the two groups were printed on sheets of different color.

In the case of asthma, the key pillars of diagnosis are the clinical symptoms, the physical examination, the breath test and the allergy status. In our empirical study we will use from among the dynamic air volumes one of the most often measured parameter, the FEV_1_^1^ value. According to the latest GINA recommendations it is not recommended for deciding on therapeutic changes in the course of ongoing asthmatic treatments, nevertheless, it preserved its value as a rating in the cross sectional analysis of asthma patient groups in which the patients are not prescribed regular inhalation treatment (or do not receive such treatment regularly). According to the latest GINA recommendation, the purpose of asthma treatment is to achieve control of the disease and to maintain such control, thus clinical control should be the basis for treatment and not the severity of the disease. The table measuring the level of control over asthma and reflecting the current approach is contained in the Appendix A.

The role of spirometry is greater in COPD than in asthma. The diagnosis and the current severity and prognosis are based on the FEV_1_ measurement. FEV_1_ measurement is a reliable and accepted method for evaluating airway obstruction. (For rating severity, here again we will use the percentage value relative to the “required” value).

The structure of the questionnaire followed the categories of the “Health Belief Model.” The model has been used as the theoretical framework, the topics of the questionnaire are following the categories of the model. Some categories contain validated multi-item scales, some are described by statements and questions based on the results of own expert interviews. The validated scales are based on Asthma Control Test, the test used in this study can be found in the Appendix B. The Asthma Control Test aims to reflect the multidimensional nature of asthma control and to demonstrate the performance against criterion measures of asthma control (Nathan et al., 2004). The asthma control test applied to our database showed a high level of internal validity (Cronbach alpha higher than 0.8).

Data gathering and field work were performed together between November 2010 and March 2011.

The research consisted of two parts:

In the first part (primary research) we carried to and distributed the questionnaires in the six lung centers, outpatient clinics where we previously had discussed with the head physician the planned research work and where agreement was also given to the conducting of such research. In some places the healthcare professionals (assistants) and the head physician distributed the questionnaires among the locally registered and treated asthmatic and COPD patients. Patients reported at the outpatient clinic spontaneously, not in response to a call. Questionnaires were filled voluntarily while waiting in the clinic.

The completed questionnaires could be assigned to the source documents used by codes.

The studied variables recorded from the source documents were these:

- date (year) of diagnosing the disease
- spirometry parameter (FEV_1_)
- treatment, medication prescribed by the treatment physician.

The comparison enabled to control the answers of the self-administered survey with the registered data of the patient dossiers and in some cases with the information of doctors.

## Results

The data are analyzed and the results are presented in a grouping matching the variants of the Health Belief Model, regarding the asthmatic and the COPD patients, including a comparison of the two patient groups.

The analysis of data has been done with using mainly univariate and bivariate statistical methods and for some variables multivariate methods as well. We investigated the means and standard deviations for the description of the variables and categories. For the analysis of relationship we used the cross-tabulation with Chi-square test, the variance analysis (ANOVA) and the Pearson correlation and linear regression models. For the comparison of perceived data, mentioned in the self-administered survey and the registered patient data we used the comparison assigned into categories “matching” and “perception is better” or “perception is worst.”

### The socio-demographic status of respondents

The sample consists of 163 asthmatic respondents and 147 COPD-respondents. The distribution according to the gender of respondents is not the same in the 2 sub-samples, in the COPD sample there is no difference between the number of male and female respondents, in the asthma sample the number of female respondents is the double of the male respondents.

The 2 sub-samples are different according to the qualification as well: in the COPD sub-sample there are significantly more respondents with lower qualification degree.

In the distribution according to age there are significant differences as well: the number of COPD-respondents above 61 years is approximately the double of the asthma-patients in the same age category, and in the age category under 40 there are only a very few COPD-patients.

In accordance with the age distribution of the respondents there are significantly more patients retired among the COPD-patients (Table 1).

**Table 1:** **Distribution of sample according to the socio-demographic characters of patients (number)**.

	**Asthma-patients**	**COPD-patients**
**GENDER**		
Male	54	74
Female	109	73
**QUALIFICATION**		
Univ., college degree	26	19
High school degree	65	38
Skilled worker	44	56
Primary school	25	34
**AGE**		
–40 years	44	4
41–60 years	84	71
61– years	35	72
**PROFESSION**		
Professionals	76	29
Retired	87	118

### Perceived susceptibility

Fifty-one percent of the asthmatic patients have been aware of their disease for more than 10 years, 21% of them between 5 and 10 years and 28% have been aware of their asthma for less than 5 years. A comparison of the “patient date” indicated by the patient and the date of the first asthma or COPD diagnosis as found in the source document, i.e., the “control date” has revealed that the patient and control dates matched in the ratio of 31.2%, while 60.3% of the patients have known about their asthmatic disease from earlier, while in 8.5% of the cases the patient perceived time was shorter than that suggested by the control date. Forty-five percent of the COPD patients have been aware of their disease for <5 years, 26% of them between 5 and 10 years and 29% have been aware of their COPD for more than 10 years. When comparing the patient and control data, the two dates matched for 32.2% of the patients, and this ratio is very similar to that of the asthmatic patients. Nearly 20% of the patients became aware of their disease later than the control date and this ratio is twice as high as in the case of asthmatic patients. Nearly 48% of the COPD patients dated their disease earlier than the control date.

A comparison of the “patient date” indicated by the patient and the control diagnosis date reveals that 53.2% of asthmatic patient started visiting a specialist later than the diagnosis date, and in around 28% of the cases did the perceived and the control date match. In the case of COPD patients, the perceived and the control dates matched in the ratio of 31%, while for 46% of them the difference showed that they had started visiting a specialist earlier than when the disease was diagnosed.

### Perceived severity

The patients rated their own condition by answering the questions of the Asthma Control Test (in the case of COPD the use of the asthma control test was arbitrary, as the application of the test is not so much recognized as justified as in the case of asthma).

The observable results of a comparison of the subjective judgment about the condition with the FEV data (Table 2) kept on the case-sheets have confirmed those previous international and Hungarian studies that found that 40–60% of asthmatic patients were not controlled (here 63.4%). According to the results of this survey altogether 7 patients said that their disease was controlled. Among them in 6 cases the LF parameter also “confirmed” this (mild asthma). Among the 36 partly controlled patients the asthma was categorized as mild for 22 and moderate for 12 based on the LF. Remarkably, among the patients who considered their disease not controlled (altogether 85 of them) the distribution of mild-moderate-severe cases was 1/3–1/3–1/3. As can be seen, 90% of all patients with severe asthma considered their disease not controlled. This is not surprising. On the other hand, however, approximately half of all mild asthmatic patients think that their disease is not controlled. 34% of them said it was partly controlled and 9% considered their asthma controlled, that is held in balance.

**Table 2:** **The perceived and observed condition in asthma and COPD**.

Absolute value and %^*^	ASTHMA^**^				**COPD**			
	**OBSERVED**				**OBSERVED**			
**PERCEIVED**	**Mild**	**Moderate**	**Severe**	**Total**	**Mild**	**Moderate**	**Severe**	**Total**
**Controlled**	6	1	0	7	1	1	0	2
	4.5	0.7	0	5.2	0.8	0.8	0	1.6
**Partly controlled**	22	12	2	36	8	14	1	23
	16.4	9	1.5	26.9	6.4	11.2	0.8	18.4
**Not controlled**	33	22	30	85	30	43	21	94
	24.6	16.4	22.4	63.4	24	34.4	16.8	75.2

* *Number and percentages of respondents*.

** *In the case of asthmatic patient the correlation analysis with Chi square test was significant at 1% level of significance*.

The number of not controlled patients is higher among the COPD patients (75%) and only 1.6% (2 persons) feel their disease is fully controlled (true, they are not suffering from severe COPD according to their breath function tests). Among the not controlled patients the mild-moderate-severe COPD patients are distributed almost equally. 84% of all severe COPD patients are not controlled according to patient judgments (which is not surprising), however, 75% of mild rated COPD patients also consider their disease uncontrolled.

### Perceived benefits

Moving on to the questions related to the treatment and the usefulness of the treatment, a comparison of the patient-listed data pertaining to medical treatment with the control data gives us a picture about whether the patient knows clearly the treatment prescribed for him/her. In 84% of the cases the data of the two sources matched, in other words the majority of the patients knew correctly the products that are to be taken (naturally, by this the patient did not say that he/she took all of them regularly according to the prescription).

It seems from responses of asthmatic patients that most of the patients know exactly why they need to take a certain medicine, trust the treatment and judge it useful.

The previously obtained data that 63% of the patients do not consider their asthmatic disease controlled or balanced contradicts this result. Thus, something is not all right here. The medicines are good, advanced, effective and the patients know them, yet it seems that in the longer run they do not consider them sufficient for bringing about long-term comfort. Long-term comfort requires something more, i.e., a more active, more conscious participation, changing of the lifestyle (attitude), which cannot be achieved always (35% of them admits not having succeeded in observing all of these despite all the good intentions).

The same comparison in the case of COPD patients shows that 94% of the patients knows exactly the type of medicine he/she takes (or should take), which corresponds to the similarly high ratio found in the case of asthmatic patients.

Half of the patients feel that they are perfectly effective and the other half thinks the medicines they take are not perfectly effective. From the medical perspective these responses seem absolutely rational, and correlate well with the nature of this disease, because they show that COPD is an irreversible, progressing disease, in which even the best medicines can only alleviate the symptoms and the complaints, but cannot fully eliminate the basic disease, i.e., the constriction of the airway.

Like in the case of asthmatic patients, here again the willpower is insufficient for executing the lifestyle and way-of-living changes proposed by the treatment physician.

### Perceived barriers

The responses to this group of questions point out the gaps in therapy-related adherence. What we have known from other studies were confirmed in this one: approximately half of the asthmatic patients sometimes miss a medicine (medicines) and mainly because they forget to take it/them. Fear of addiction or side effects and neglecting the treatments because of this are not common.

It seems that patients stick to the habit of meeting the treatment physician regularly. They do not forget this, because this is a single, infrequently recurring obligation that is easier to observe than other requirements that (would) need to be fulfilled in the long run, regularly, under any circumstances (e.g., medication).

Those who do not find regular check-up visits difficult at all represent a high percentage. This high “willingness” could be explained by the fact that 60% of the respondents were pensioners. As a notable fact, relatively many of the respondents mentioned long waiting in the outpatient clinic as an unfavorable factor that may detain them from regularly visiting their specialist.

In this group of questions—that is in relation with adherence to therapy and the barriers to it—similar results have been found in the case of COPD patients, as in the case of asthmatic patients: sometimes the COPD patients neglect taking their medicines and many of them because they forget.

The number of those who see no barriers and difficulties at all in going for check-ups occasionally is high, but this is not accidental. The percentage of retired people is even higher (80%) among these respondents.

### Cues to actions

It can be said of the asthmatic patients that—as they admit—they use their inhalators with convincing ease (92% of the asthmatic respondents and 90% of the COPD respondents told they are sure they use the inhalators in the correct way). Similarly self-confident responses were given regarding the familiarity with and effectiveness of the medicine (92% of the asthmatic respondents and 90% of the COPD respondents told they are familiar with the effects of the medicines). The question is raised how the correct use of the inhalator and the efficacy of the product are connected in the thinking of the patient. Is the patient aware that the correct use of the inhalator is naturally a pre-condition for product efficacy, but at the same time it may happen that despite correct use of the inhalator the product is insufficient or ineffective for the patient?

It is not typical of the patients asked that they want to consult (about 50% of the patients in both diseases) discuss with the doctor the disease, the “empowered patient” is not common. Paternalism, convenient, passive reliance on the treatment physician is powerfully present, so much that the patient expects information mainly from the doctor. This attitude may be related to the type of health care system in which the patients were socialized. For asthmatic patient, Internet plays an important role in information gathering (Table 3).

**Table 3:** **Information sources and commitment of patients**.

**% (*n*)**	**Asthma**	**COPD**
**ARE YOU INTERESTED IN THE LATEST RESEARCH RESULTS?**		
Extremely interested	19 (30)	20 (29)
Very interested	13 (20)	19 (27)
Interested	57 (91)	49 (69)
Not very interested	11 (20)	12 (20)
**HOW DO YOU OBTAIN INFORMATION?**		
Personally from the treatment physician	40 (100)	64 (116)
At organized meetings/in the club	2 (5)	3 (5)
From the Internet	22 (56)	8 (15)
From flyers	11 (26)	8 (15)
From television, radio, newspaper	25 (63)	17 (32)

COPD patients also state with convincing self-confidence that they use their inhalators correctly. Paternalism is strongly present among them as well. They are interested in everything that is related to their disease, but they do not look up information independently, “proactively,” as they expect their physician to inform them on what he/she thinks is needed, because the doctor will surely know better what the correct care for the patient is.

In the case of asthmatic patients having a say in the matter of medication is more frequent, while COPD patients leave this fully to their doctor.

In comparison with the asthmatic patient group the ratio of Internet users is insignificant, thus reaching the COPD patients is not the most effective through this channel. The role of written and electronic press is not dominant, either, only the personal relationship is important. This may be related to the higher average age of the COPD patients.

It is equally surprising that (like asthmatic patients) they do not prefer patient communities in gathering information and exchanging of experience.

The extent to which asthmatic patients and COPD patients do not seek patient community participation could be quite striking. An explanation for this could be that in the given healthcare system few patient clubs can be found and the intention for organizing patient communities and self-organization is also low.

### Self efficacy

The picture is varied in terms of attitudes, forms of behavior, own efforts that the patient is willing to mobilize in order to achieve greater health. Typically, the activities that require the least effort are the most popular ones. It is much more comfortable to say that I avoid stress than to take on a form of regular exercise, although no great differences can be seen among the average values of the responses (Table 4).

**Table 4:** **Efforts to maintain health**.

	Asthma (average value)^*^	COPD (average value)^*^
Less stressful lifestyle	3.49	3.53
Gave up smoking	3.42	3.23
Healthy nutrition	3.38	3.17
Taking vitamins	3.33	3.1
Reducing smoking	3.27	3.06
Controlled body weight	3.15	3.03
Exercise	3.06	2.88
No effort	1.85	2.1

*Responses were given in a 5-point scale, where 5 meant fully agree, and 1 meant fully disagree*.

* *Significance at 0.05 level*.

In case of a deteriorating condition, the certain point is the doctor, and experimenting with other methods is not typical.

According to the literature the efforts to maintain the health or level of condition of asthma or COPD have a correlation with socio-demographic variables only in a few cases and do not have any correlation in the most cases.

Regarding the gender of the respondents there are some differences between the attitude of men and women, but the relationship is significant only for taking vitamins (women take vitamins according to more respondents) and for controlled body weight (women control the body weight rather than men) (Table 5).

**Table 5:** **Efforts to maintain health according to the gender of respondents**.

		**Asthma**	**COPD**
Less stressful lifestyle	Male	3.32	3.67
	Female	3.59	3.38
Gave up smoking	Male	3.30	3.39
	Female	3.50	2.70
Healthy nutrition	Male	3.20	3.12
	Female	3.49	3.23
Taking vitamins	Male	3.10	2.73^*^
	Female	3.46	3.38
Reducing smoking	Male	3.38	3.18
	Female	3.21	3.29
Controlled body weight	Male	2.90^*^	3.17
	Female	3.32	3.05
Exercise	Male	3.08	3.05
	Female	3.07	2.72
No effort	Male	2.00	2.08
	Female	1.77	2.13

* *Significant at 0.05 level*.

According to the age of the respondents there are some differences in the case of some factors, but the relationship is significant only for the less stressful lifestyle (Table 6).

**Table 6:** **Efforts to maintain health according to the age of respondents**.

		**Asthma**	**COPD**
Less stressful lifestyle	−40 years	3.08^*^	^**^
	41–60 years	3.70	3.44
	61– years	3.77	3.56
Gave up smoking	−40 years	3.47	
	41–60 years	3.35	2.78
	61– years	3.57	3.71
Healthy nutrition	−40 years	3.15	
	41–60 years	3.53	3.26
	61– years	3.48	3.12
Taking vitamins	−40 years	3.54	
	41–60 years	3.24	3.00
	61– years	3.15	3.14
Reducing smoking	−40 years	3.58	
	41–60 years	3.00	3.50
	61– years	3.24	2.94
Controlled body weight	−40 years	2.97	
	41–60 years	3.23	3.32
	61– years	3.40	2.95
Exercise	−40 years	2.94	
	41–60 years	3.13	2.78
	61– years	3.24	3.00
No effort	−40 years	1.83	
	41–60 years	1.97	2.10
	61– years	1.70	2.05

* Significant at 0.05 level,

** *Not applicable because of the low number of respondents (the total number of COPD-respondents younger than 40 years is 4)*.

The condition of disease could have an effect on the behavior how the patients try to maintain the level of disease and try to avoid the deteroriation of the condition. According to our research there are some differences in behavior depending the perceived level of asthma or COPD, but the differences are significant only in few cases (Table 7).

**Table 7:** **Efforts to maintain health according to the perceived condition of asthma or COPD of respondents**.

		**Asthma**	**COPD**
Less stressful lifestyle	Controlled	2.58^*^	3.13
	Partly controlled	3.47	3.65
	Not controlled	3.69	3.42
Gave up smoking	Controlled	4.08	2.88
	Partly controlled	3.57	3.24
	Not controlled	3.23	3.06
Healthy nutrition	Controlled	3.75	2.63
	Partly controlled	3.47	3.22
	Not controlled	3.44	3.29
Taking vitamins	Controlled	3.58	2.00
	Partly controlled	3.06	3.26
	Not controlled	3.43	3.19
Reducing smoking	Controlled	3.78	3.14
	Partly controlled	3.26	3.43
	Not controlled	3.23	2.93
Controlled body weight	Controlled	2.92	3.00
	Partly controlled	3.27	3.08
	Not controlled	3.20	3.15
Exercise	Controlled	2.50	2.38
	Partly controlled	3.21	2.97
	Not controlled	3.10	2.94
No effort	Controlled	2.00	2.50
	Partly controlled	1.71	1.90
	Not controlled	1.93	2.30

* *Significant at 0.05 level*.

## Discussion

In this study, our purpose has been to explore the factors that obstruct adherence and any implications to possible actions related to them.

### Factors and effects of perceived threat

The result that ~2/3 of the patients have been aware of their asthmatic disease and close to half of them of their COPD from earlier than the control date raises the question what the patient really considers as a disease, what he/she perceives as a disease. It might be that patients regard as the start of their disease the beginning of their allergic symptoms “only” that preceded the disease.

Another problem is due to the fact that the source document contains the first diagnosis in adulthood, which might be a source of error if the patient was already diagnosed with asthma in childhood.

The difference could be explained by a lengthy patient journey, too: the patient might visit several doctors between the first symptoms and the setting up of the diagnosis, until he/she finally arrives at the pulmonary center. For instance, a COPD patient described that he/she visited several places starting from the general practitioner, through laryngology and up to allergy examination for years with his/her coughing symptoms (coming off with negative results from everywhere), until he/she accidentally visited the lung center, where the disease was finally diagnosed and the patient was taken into care.

More thought-provoking is the fact that half of the patients began visiting a specialist later than the date of the first diagnosis that we know. It may be that as much as even half of the patients become seriously aware of their disease only later.

One might look for reasons in the players themselves. The question may be asked how much emphasis was laid by the specialist on sharing with the patients openly and in details the essentials, nature, progress, expected consequences, perspectives and treatment options of the disease at the time of setting up the diagnosis. Given the statistics of patient turnover and the lack of professionals one may guess that there are gaps in the doctors' information sharing.

But when the data are examined from the aspect of the patient, it seems as if the setting up of the diagnosis would only be a single event for the patient, without any consequences (even if medication therapy has been ordered). It is a known fact that asthmatic patients report their symptoms early (unlike the COPD patients), because the first symptom in most cases is an asthmatic attack, which is frightful for a lay person and the patient him/herself experiences it as scary. When the patient receives help, although diagnosis is given, if the patient is fully restored to health and can perform daily activities without complaints, then his/her faith will be strengthened that this attack was in fact a one-time event, just a bad memory. The patient will typically accept the disease when the problems reappear.

Thus, as regards perceived susceptibility and the acceptance of the diagnosis, one may conclude based on responses given to this group of questions that it takes a long time for the patient after the appearance of the first complaints to get to a specialist, get and accept an accurate diagnosis, and accept the necessity of regular treatment, check-ups, and follow-up. It is difficult for the patient to accept that suddenly a change occurs in his/her usual lifestyle and capabilities, which forces him/her to reconsider his/her work, lifestyle, his/her responsibility for him/herself and his/her environment and the sources of risk that should be avoided. This behavior is typical in general in the case of chronic diseases.

However, it seems that once the patient got to the end of this long journey (of recognition and acceptance), he/she will see his/her limitations clearly, together with the causes and maintainability of the disease.

Once the disease is accepted, the patients will understand what they can expect, and more or less become familiar with their disease, the sources of risk to be avoided, and learn what to do when a problem arises. They assess and understand that they have to and can live with this disease. There will be better and worse periods in the progression of the disease, but living with the disease can be made easier by the new “rules of the game,” changes in the way of life and the lifestyle.

The fact that half of the patients felt the balancedness of their disease was worse than what their actual breath function results would suggest, could be explained by the fact that these patients experience dyspnea episodes more often than the average, they have complaints more often (perhaps due to irregularity in following the therapy), or they have not accepted the changing of their lifestyle yet. It may be concluded from this that they use emergency (bronchodilator) medicine more often, which “improves” the control value, while the subjective value (showing the experiences and observations of the past one month) remains unchanged.

This implies that closer, more frequent or rather more thorough contacts are necessary in order to enable the treatment physician to control correct adherence to therapy.

### Factors and effects of perceived benefits and barriers

The group of patients we asked understand and know what they should use and why in order to keep their disease in balance and consider themselves self-confident in this knowledge. The patient is content with the medicine products, because they are received ready-made. The medicine has an exact dosage, and the patient needs not make special effort to observe the recommended administration frequency and dose. However, the modern product is insufficient for real management and keeping in balance of the disease. Nevertheless, as soon as changes in the lifestyle need to be made, we immediately see serious gaps in adherence.

Half of the patients forget to take the medicine sometimes, we cannot know how long the medicine is missed, nevertheless, it is clear that there are adherence gaps in the taking of the medicine.

### Factors of cues to action

Strong (paternalistic) attachment to the treatment physician: the patient accepts everything that the doctor says and suggests, the patient will not ask or object, he/she will not have a say in the treatment as prescribed by the doctor. In addition to powerful attachment, there is a strong reliance, too, as the patient will visit the doctor without delay in case of a problem (the patient will visit the doctor and not look for other ways) and will expect all information to be given personally by the doctor. This result shows the importance of the regular contacts with the doctors, but it shows also that the patients are expecting much help from the doctor.

The patients expect information on everything, but inconsistently they entrust all decisions related to their care to their doctor, as the patient cannot understand everything, anyway.

Patients claim they are interested in everything that is related to their health and disease, yet the sources of obtaining the information vary greatly. They consider the doctor to be the surest source of information, that is why patient education by the doctor is so very important.

Although the patient often feels abandoned, the self-organized patient groups and patient clubs, if any, carry little significance, as patients are less interested in participating. This is partly associated with the general patient culture and also the local healthcare system.

One reason for the self-organized patient communities could be to teach patients how to ask questions, how to argue (in the right sense of the word) with the treatment physician, so that all problems and fears—that might induce poor adherence—come to the surface.

Explaining and learning to ask—the physician has also something to learn in this area. There is high need for simple explanations, and the doctor should explain to the patient what is useful and why and what needs to be done, by showing levels of cause and effect. In addition to information sharing, it is very important also to check if the patient really understood what is to be done.

### Factors of self efficacy

The responses given to the last question in this group are rather contradictory. The patients request (and expect) full information, because they feel this is how they can participate responsibly in their healing, but at the same time they fully rely on the doctor (and so paternalism will prevail in the end).

This is thought-provoking also because although the patient expects information sharing as a passive process whereby a set of information is poured into his/her head, which is not up to him/her to initiate, if possible, but it is best done by the treatment physician on his/her own initiative.

The attitude analysis revealed similar characteristics in the case of the COPD patients, too.

The patients are more willing to move—at least initially—in the direction of actions that require less energy invested. It is easier to say that they strive for what is good and healthy, but regular exercise is not a popular choice (although according to the latest scientific developments a combination of pharmacotherapy and exercise training for respiratory rehabilitation is a real breakthrough in the treatment of COPD).

Attachment to the treatment physician is powerful among the COPD patients, too; he/she is contacted and sought when there is a problem. Anticipation of full information from the doctor and reliance on the doctor are apparently opposing notions, but in my opinion, like in the case of asthmatic patients, here again the patient expects passive information receiving and not a joint decision or agreement based on discussion.

In respect of the cues to action and self-efficacy as analyzed in the model, in other words in respect of what those suffering in these two chronic diseases are willing to carry out for the sake of their own health, no significant correlation can be demonstrated according to the demographic variants. The results are similar in the literature, and where a correlation is manifested with the demographic variants, this correlation is still unclear. (Howell, 2008) in his research shows a difference between the sexes, such as the women are more inclined to non-adherence, but this difference disappears with age (Lemmens, 2009).

Non-adherence is not significantly related to the type or severity of disease, with rates of between 25 and 30% noted across 17 disease conditions (DiMatteo, 2004).

## Conclusions

Our aim was to analyse the factors of health behavior influencing patient adherence in asthma and COPD. We can confirm the results in the literature according to the few relationships among socio-demographic variables and behavioral variables and the factors of adherence. Because of the limitations of the research the results and the possible conclusions have explorative character.

### Conflict of interest statement

The author declares that the research was conducted in the absence of any commercial or financial relationships that could be construed as a potential conflict of interest.

## Footnotes

^1^FEV_1_ (Forced Expiratory Ventilation) expresses in litres the volume of exhaled air in the first second of a forceful exhalation started right after a maximum inhalation. This value can also be expressed as a percentage, if (in standardized tables) we look at where the currently measured value is positioned (how many %) in comparison with the so-called “required” (or “predicted normal”) value corresponding to the patient's age, sex and height. This percentage FEV_1_value (that is the FEV_1_value expressed as a ratio of the “required” value) is used in the assessment of the clinical severity of asthma.

## Appendix A

**Table A1:** **Levels of asthma control**.

**Characteristic**	**Controlled**	**Partly controlled**	**Uncontrolled**
Daytime symptoms	None, twice or less/week	More than twice /week	Three or more features of partly controlled asthma
Limitation of activities	None	Any	
Nocturnal symptoms/awakening	None	Any	
Need for reliever/rescue treatment	None/twice or less/week	More than twice/week	
Lung function (PEF or FEV_1_)	Normal	<80% (predicted or personal best, if known)	
Exacerbation	None	>1/year	

**Table A2:** **Classification of severity of airflow limitation in COPD (based on post-Bronchodilator FEV_1_)**.

**GOLD 1 Mild**	**GOLD 2 Moderate**	**GOLD 3 Severe**	**GOLD 4 Very Severe**
FEV_1_ > 80% predicted	50% < FEV_1_ < 80% predicted	30% < FEV_1_ < 50% predicted	FEV_1_ < 30% predicted

## Appendix B

Asthma Control Test™

The ASTHM A CONTROL TEST™ is a quick test for people with asthma 12 years and older. It provides a numerical score to help assess asthma control.

INSTRUCTIONS:

1. Write the number of each answer in the score box provided.
2. Add up the score boxes to get the TOTAL.
3. Discuss your results with your doctor.
  Name:                     Today's Date:

1. In the past 4 weeks, how much of time did your asthma keep you from getting as much done at work, school or at home?
  SCORE … …
  All of the time [1]
  Most of the time [2]
  Some of the time [3]
  A little of the time [4]
  None of the time [5]
2. During the past 4 weeks, how often have you had shortness of breath?
  SCORE … …
  4 or more nights a week [1]
  2 or 3 nights a week [2]
  Once a week [3]
  Once or twice [4]
  Not at all [5]
3. During the past 4 weeks, how often did your asthma symptoms (wheezing, coughing, shortness of breath, chest tightness or pain) wake you up at night or earlier than usual in the morning?
  SCORE … …
  4 or more times per day [1]
  1 or 2 times per day [2]
  2 or 3 times per week [3]
  Once a week or less [4]
  Not at all [5]
4. During the past 4 weeks, how often have you used your rescue inhaler or nebulizer medication (such as albuterol)?
  SCORE … …
  3 or more times per day [1]
  1 or 2 times per day [2]
  2 or 3 times per week [3]
  Once a week or less [4]
  Not at all [5]
5. How would you rate your asthma control during the past 4 weeks?
  SCORE … …
  Not controlled at all [1]
  Poorly controlled [2]
  Somewhat controlled [3]
  Well controlled [4]
  Completely controlled [5]
  If your score is 19 or less, your asthma may not be as well controlled as it could be.
  No matter what your score is, share the results with your healthcare provider.
  TOTAL:……
