# Supplementary material for: Attitudes of Hungarian asthmatic and COPD patients affecting disease control: empirical research based on Health Belief Model
Source: Front Pharmacol. 2013 Nov 13;4:135. doi: 10.3389/fphar.2013.00135 (PMC3826076; doi:10.3389/fphar.2013.00135)
Supplement: Supplementary file 1 [file Presentation1.PDF]

# Attitudes of Hungarian asthmatic and COPD patients affecting disease control: empirical research based on Health Belief Model

**Judit Simon,** Corvinus University of Budapest, Institute of Marketing and Media, Health Marketing Research Centre, 1093 Budapest, Fővám tér 8., judit.simon@uni-corvinus.hu

**Introduction:** Patient non-adherence to treatment is a major problem across most chronic diseases. In COPD and asthma treatments it is a complex issue because people need to make behavioural and lifestyle changes while taking medications. Poor adherence results in increased rates of morbidity and mortality, more frequent hospitalisations, and ultimately higher healthcare expenditures.
**Material and methods:** The objective of the study was to assess asthmatic and COPD patient's attitudes toward adherence in Hungary. Health Belief Model was used to help explain reasons of non-adherence. The results of the study should provide additional support to understanding health-related behaviours and to developing health related programs enhancing adherence of asthmatic and COPD patients.
145 diagnosed COPD patients and 161 diagnosed asthmatic patients were involved in 6 pulmonary centres. The questions were designed to measure Health Belief Model dimensions A 1-5 point verbal Likert scale was used. As a second stage, the answers were compared with the registered patient's personal health data available in pulmonary centre's documentation. The data was analysed using SPSS software.
**Results:** More than 32% of patients are very interested in new asthma or COPD research results, but their main information source is physician. The trust toward the physician is very high. Patients accept treatments and rarely ask questions. Respondents are cooperative but sometimes fail to follow therapeutic recommendations. There is no willingness to join self-help groups or associations.
**Discussion:** The paternalistic approach was generally accepted, moreover expected by the patients from the physicians. It is important to train patients, increase their self-efficacy, responsibility and involve them into self-management programs. Both physicians and patients should be trained how to communicate – this approach can lead to increased understanding and better adherence.

**Keywords:** medication adherence, chronic diseases, health belief model, asthma, COPD



MAGYARORSZÁG MEGÚJUL
A projekt az Európai Unió támogatásával, az Európai Szociális Alap társfinanszírozásával valósul meg.
